# Illuminating Perspectives: Navigating Eye Care Access in Sub-Saharan Africa Through the Social Determinants of Health

**DOI:** 10.7759/cureus.61841

**Published:** 2024-06-06

**Authors:** Mam Jarra Gai, Varsha Reddy, Vivian Xu, Neda H Noori, Michelle Demory Beckler

**Affiliations:** 1 Dr. Kiran C. Patel College of Osteopathic Medicine, Nova Southeastern University, Fort Lauderdale, USA; 2 College of Osteopathic Medicine, Michigan State University College of Osteopathic Medicine, East Lansing, USA; 3 Microbiology and Immunology, Dr. Kiran C. Patel College of Allopathic Medicine, Nova Southeastern University, Fort Lauderdale, USA

**Keywords:** healthcare policy, healthy equity, access to eye care, public health education, global vision, global public health, sub-sahara africa, social determinants of health (sdoh), eyecare

## Abstract

Ensuring access to proper eye health services is not only a fundamental human right but also crucial for preserving an individual's quality of life, preventing blindness, and promoting overall well-being. This is especially true in low-income countries like Sub-Saharan Africa (SSA) where recognizing the intricate relationship between access to healthcare and social determinants of health (SDOH ) is crucial to addressing health disparities. The goal of this study was to elucidate and highlight not only the barriers millions face in obtaining eye care but also pave the way for interventions and policies aimed at creating equitable access across diverse populations. To do this, a scoping review was conducted across the Cumulated Index to Nursing and Allied Health Literature (CINAHL), Embase, and PubMed databases for studies meeting the search terms and inclusion criteria. The results show that intervention strategies that increase vision care must extend beyond the healthcare sector to address the multifaceted challenges. Collaborating with stakeholders involved in addressing broader livelihood issues, such as food security, education, and SDOH, becomes imperative to ensure comprehensive and sustainable improvements in vision care accessibility in SSA.

## Introduction and background

Eye care remains a significant area of unmet need globally, with an estimated 2.2 billion individuals affected by vision impairment, particularly in Sub-Saharan Africa (SSA), where disparities in access and delivery to eye care are pronounced [1,2]. The multifaceted nature of this issue encompasses various factors such as geographical location, gender, socioeconomic status, and literacy levels, all influencing the utilization of vision care services [3,4]. Access to adequate vision health not only improves individual well-being but also contributes to reducing healthcare disparities [5,6]. However, achieving good vision health faces considerable challenges despite the economic burden associated with vision loss far exceeding the costs of addressing impairment, underscoring the urgency of enhancing access to eye care services [7].

In SSA, approximately 18-25% of the population grapples with eye diseases, exacerbating the region's healthcare challenges [8]. Avoidable visual impairment and blindness remain pervasive across the region [1,2]. The factors leading to eye diseases and their treatment outcomes can often be traced back to social determinants of health (SDOH) [6]. According to the World Health Organization (WHO), SDOH encompasses the conditions under which individuals are born, grow, live, work, and age [9]. These circumstances are influenced by the distribution of wealth, authority, and resources at various levels, including global, regional, and local scales. Addressing the fundamental effects of SDOH is crucial because individuals are more inclined to achieve improved health outcomes when they have access to resources such as quality education, secure housing, safe surroundings, and adequate healthcare coverage [6,8,9,10].

In the context of access to vision care in SSA, limited access is exacerbated by factors like the shortage of skilled healthcare workers, low health literacy levels, and insufficient prioritization of vision health [8,11]. Societal factors, including policies and local practices, significantly influence how vision care is delivered and contribute to the disparities in access to eye care [7,11]. Addressing the systemic barriers and tailoring the needs of individual communities is crucial for improving eye care access and reducing inequities in SSA and beyond.

This research paper aims to delve into the complexities of navigating eye care access in SSA, particularly through the lens of SDOH, to identify strategies for enhancing access to eye care services and ultimately improving vision health outcomes in the region.

## Review

Methods

This scoping review followed the methods outlined by Arksey and O'Malley [12] as well as Levac et al. [13]. The review process involved distinct phases including formulating the research question, identifying pertinent studies, selecting studies that met the set inclusion criteria, organizing and extracting data, and synthesizing and presenting the findings.

Eligibility Criteria

To meet the eligibility criteria, original peer-reviewed research articles must have been published in English between 2013 and 2023. Acceptable article types included systematic reviews, meta-analyses, observational studies, reports as well as gray literature, including government reports and organizational documents that captured a comprehensive view. Non-peer-reviewed articles, editorials, opinion pieces, review papers, and letters were excluded. To be included, studies must also specifically address limitations in access to eye care services in individuals of all ages in SSA. The Preferred Reporting Items for Systematic Reviews and Meta-Analyses (PRISMA) flowchart [14] was employed to organize the inclusion process.

Information Sources and Search Strategy

**Figure 1:**
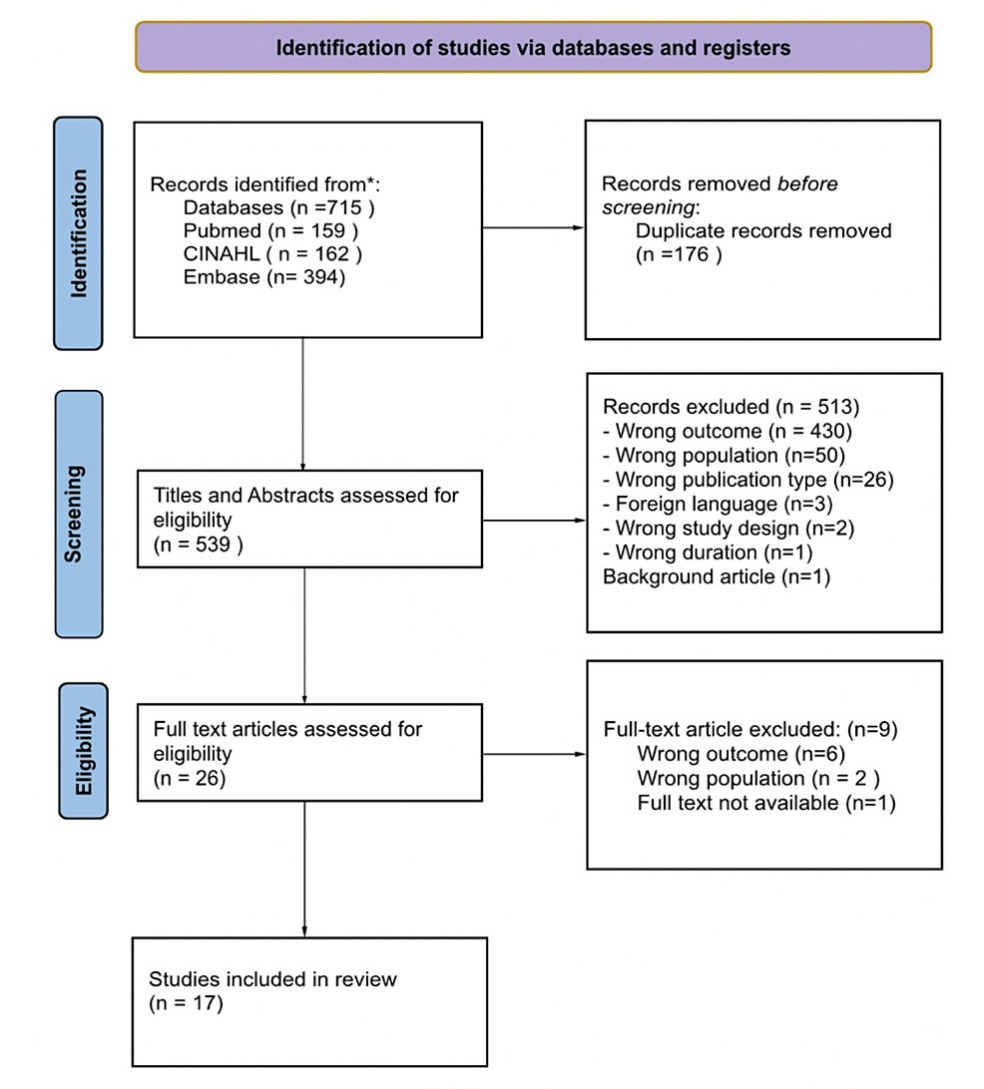
The PRISMA flowchart delineates the study selection process. CINAHL: Cumulated Index to Nursing and Allied Health Literature The Preferred Reporting Items for Systematic Reviews and Meta-Analyses (PRISMA) flow diagram illustrates the stages of literature search, screening, and eventual inclusion of studies in the review.

Access to articles was sought through electronic databases such as CINAHL via EBSCOhost, Embase, and PubMed. The search terms employed variations of "eye" or "vision care," "social determinants of health," and specific countries within SSA. The primary reviewer conducted the initial search, focusing on articles related to limitations to accessing eye care in SSA and the associated SDOH. The search terms included terms relevant to the topic including a list of countries in SSA obtained from The InterTASC Information Specialists' Sub-Group Search Filter Resource [15]. These terms were applied as controlled descriptors in CINAHL, PubMed, and Embase databases. The Boolean operator "AND" was utilized for simultaneous occurrences, while "OR" was used for synonyms. The specific search terms utilized are outlined in the PRISMA flow diagram seen in Figure 1.

Study Selection Process and Quality Assessment.

The initial search yielded 715 publications. After eliminating 176 duplicates, 539 articles remained for relevance assessment. Each title and abstract were screened by all four authors. Full-text articles meeting the initial screening criteria underwent further review. Any discrepancies were resolved through consensus, resulting in 17 studies for further analysis.

Data Collection Process and Synthesis

A digital spreadsheet template was used for data extraction from eligible articles. Each reviewer independently abstracted data from the articles included. Individual templates were merged into a master spreadsheet for collective analysis. Summary outcomes and discrepancies were discussed among reviewers to reach a consensus. Extracted data included study details, objectives, findings, recommendations, and limitations. Reviewers synthesized data into relevant groups, which were later translated into the results section by two reviewers. The primary reviewer reviewed and finalized the draft. Throughout the revision process, all four reviewers addressed discrepancies to reach a consensus.

Results 

Cost of Eye Care

Across all the studies reviewed, the cost of eye care was one of the most frequently cited barriers to accessing health services [16-23]. Cost of care was a recurring problem in rural, suburban, and urban communities. This was particularly striking in rural communities, where lack of eye care facilities is coupled with difficulty obtaining transportation to seek care. Fifteen percent of participants in Thompson et al.’s [22] study stated that distance to services was a major barrier to accessing refractive services. Poor road infrastructure is another common barrier to access [23]. In one study, living close to an eye facility was associated with an increased likelihood of utilizing services [24].

Demographic Variations in Attitudes

Attitudes toward seeking eye care services vary across different demographics. Older age was often positively associated with seeking eye care services [24,25]. Men were more likely to seek care than women [24,26]. However, in the Onyiaorah et al. [19] study surveying traders at a rural Nigerian market, females were more likely to seek care, and traders older than 50 were less likely to seek care.

Health Education and Cultural Beliefs

Several studies demonstrated that health education and cultural beliefs play a major role in how and whether people seek eye care. Lack of knowledge of eye diseases and perception of vision issues were two of the major barriers reported at three clinics in Tanzania [27]. A study found that in a rural Nigerian population, the belief that the eye disease was not serious, that aging has no cure, and a preference for spiritual treatments were all reasons not to consult an ophthalmologist [16]. In a survey conducted in a rural Nigerian community, it was found that the belief that eye ailments are not serious often stopped male patients from seeking orthodox eye care [19]. Two studies in Nampula province, Mozambique, also showed education-related barriers to eye care. Of the participants interviewed by Thompson et al. [22], 20% did not feel that their problem was severe enough to seek treatment. In Sengo et al.’s [21] study, 20.5% cited choosing to self-medicate, 17.8% cited choosing to seek traditional treatments, and 11.6% cited choosing to buy eyeglasses on the street as one of their top barriers to accessing eye health services. Lack of awareness was also cited by 31.8% of participants in Arinze et al.’s [18] survey of adults in Abagana. Having a personal history or family and relatives with eye disorders, on the other hand, was positively associated with seeking eye care [18,24,25].

Pediatric Eye Care and Education

Two of the studies reviewed focused specifically on pediatric eye care and access in schools. Yashadhana et al. [20] found that in primary schools in the central region of Malawi, visual acuity testing was not performed frequently enough, or even performed at all. There was distrust of medical providers and a general lack of education on the importance of eye care. This was further complicated by the cost of obtaining glasses as well as the stigma children face from their peers. Sukati et al. [28] found that teachers often recognized the importance of eye care but lacked knowledge about specific eye conditions or how to recognize and manage children with vision problems.

Institutional Barriers

From an institutional perspective, a shortage of medical equipment, assistive devices, and medical personnel all hinder access to eye care [29-32]. In Swaziland specifically, there are no medical schools in the country, and therefore, they must rely on healthcare providers trained in neighboring countries [29]. Several studies cited the heavy burden on tertiary care facilities. A lack of basic eye healthcare at the primary level, coupled with disorganized record-keeping, mismanagement of funding, and poor referral systems means that tertiary care centers are faced with large volumes of patients they are unequipped to handle [20,29]. This in turn contributes to the crowding in hospitals and long wait times that deter patients from seeking proper eye care [17,21].

Discussion

There remains a significant deficiency in eye care worldwide, namely, in SSA. There is a plethora of barriers to care in this region, including but not limited to misconceptions and barriers to knowledge and understanding of eye health, access to healthcare facilities, and transportation. SDOH plays a large role in the access to and utilization of eye care by this population, and this study sought to determine existing barriers through a scoping review of literature analyzing eye care status in SSA countries. 

Posing a significant hindrance to eye care seeking and utilization are certain attitudes, beliefs, and conceptions of this population regarding general eye health. A study by Olatunji et al. demonstrated certain perceptions regarding blindness and illustrated this concept. Participants surveyed identified consumption of certain foods, supernatural forces, and ages as common causes of blindness [33]. Further, individuals in this population may prefer to initially seek care from traditional healers rather than an eye care provider or medical doctor, and 65% of participants used a form of traditional eye medication [33]. Teachers can play an important role in raising awareness about eye health starting at an early age. In children who attend school, their teachers may be the first to notice vision changes such as difficulty seeing the board, inability to concentrate, proximity of books to the eyes, and squinting [34]. They may also be children’s first source of learning about eye care, and the benefits of this education can extend to the families of these children as they mature. However, these effects may be limited within areas with low school attendance or low education levels.

Poverty is a continuing obstacle to obtaining eye care, with challenges in the availability of facilities and transportation contributing to reluctance to seek eye care. Blindness is associated with poverty in some SSA countries, reflecting limited resources and lower rates of care [35]. The glaring lack of resources highlights the importance of increasing accessibility to eye care, especially in regions where inequity in healthcare exists and is compounded by the impact of SDOH.

Systems implemented to address these barriers and increase access to eye care should engage stakeholders such as government agencies and ministries of health. These institutions may work with hospitals to increase the training of ophthalmic workers beyond ophthalmologists to support functioning eye care clinics. A study involving optometry technicians in Eritrea found patterns of low confidence in skills such as refraction, managing emergencies, and supplying spectacles [36]. In addition, integrated eye care as part of primary care is a potential resolution to hesitancy in seeking specific eye care services [37]. Additional visits to medical doctors require more frequent transportation and/or other resources that can be difficult to attain.

The conclusions from the studies are limited by the small number of studies included, the population specificity of some included studies, and the limited representation of rural populations in the included studies. Table 1 summarizes key concepts from each paper included in our study, and outlines recommendations for expanding eye care access and research limitations for future consideration. Further research is required to determine the prevalence of barriers related to SDOH, their effects, and potential solutions in promoting eye health services in SSA countries.

**Table 1:** A comparative table of the included studies highlighting the barriers to eye care, recommendations for future studies, and limitations SSA: Sub-Saharan Africa; AI: artificial intelligence

Source	Study Design	Sample Size	Study Aims/Objectives	Cited Barriers to Eye Care in SSA	Cited Recommendations for Improving Access to Eye Care in SSA	Study Limitations
Onwubiko et al. (2014) [16]	Cross-sectional study	501	To identify the initial routes to eye care within a rural community in southeastern Nigeria and to pinpoint the factors linked with these pathways	Most participants initially preferred consulting a patent medicine dealer (PMD) or an ophthalmologist for eye diseases, with few opting for prayer/spiritual consultation. Reasons for not consulting an ophthalmologist included ignorance, financial constraints, poor access to eye care services, self-assessment of the disease as nonserious, belief in incurability due to aging, and preference for spiritual treatment. Formal education significantly predicted consultation with an ophthalmologist for major eye diseases	The study recommends health education campaigns, integrating traditional healers into orthodox eye care, and promoting formal education to enhance eye health outcomes. Improving access to orthodox eye care services and addressing barriers like poor access, cost, and ignorance are crucial for encouraging professional eye care seeking	This study relied on teachers' knowledge of eye care practices and did not assess individual preferences for eye care facilities. The sample of teachers may only be representative of some Swaziland teachers and their perspectives on children's eye care utilization
Mohammed & Munsamy (2024) [17]	Cross-sectional study	1615	To investigate the use of eye care services and the factors linked to their utilization among adults residing in the Ashanti region of Ghana	Gender disparities were observed in factors such as education level, employment status, and perception of eye care importance. Distance traveled for eye care did not differ by gender. Most cited difficulty with distance vision as the primary reason for seeking care. Challenges encountered included waiting time and the cost of eye care	The study recommended empowering the National Eye Care Unit to integrate eye health into healthcare delivery and implementing measures to address barriers to eye care utilization	Constraints and exclusion of individuals under 18 years of age, highlighting the need for comprehensive pediatric eye care services
Arinze et al. (2015) [18]	Cross-sectional study	549	To determine barriers and incentives to eye care utilization	Main barriers to eye care utilization included lack of awareness (31.8%), cost (18.0%), and fatalistic attitudes (15.9%), while possessing health insurance, family history of eye disorders, noticing changes in vision, current eye disease, or systemic comorbidity were motivating factors	Educational interventions on eye health maintenance and eye health-seeking behaviors and measures to reduce eye care cost	Intrinsic bias in self-reported data and setting in a rural area
Onyiaorah et al. (2022) [19]	Cross-sectional study	177	To determine the healthcare provider first sought, reasons, and symptom duration before hospital presentation among traders in rural Nigeria for ophthalmic symptoms	The median duration before seeking care was 83 days. Reasons for not seeking orthodox eye care initially included cost, perception of ailment severity, and advice from friends. Females were more inclined to seek orthodox care, while males were more likely to perceive their ailment as nonserious. Traders over 50 years old were less likely to seek any care for eye ailments	Eye health education and cost reduction would improve the uptake of orthodox eye care services	Poor generalizability due to analysis of only a specific population
Yashadhana et al. (2023) [20]	Qualitative case study	44	Assessing the availability, accessibility, barriers, and enablers of school-based eye health programs in primary schools in the Central region of Malawi	Visual acuity testing in schools needs to be more consistent and present, leading to undiagnosed vision issues among children. Children frequently endure social stigma related to wearing glasses and facing bullying and judgment from peers. In Malawi, where 70% live rurally, transport barriers hinder eye care access, compounded by affordability issues with glasses. Trust issues with healthcare providers and low eye care awareness persist. Hospitals' disorganized record-keeping and weak referral systems strain tertiary care	Enhancing eye care access involves routine school vision screening by teachers, community-based basic eye care to reduce secondary and tertiary center burdens, upgraded training for eye care practitioners, and community health promotion and education efforts	Lack of representation of rural regions of Malawi and unequal representation between male and female participants
Sengo et al. (2022) [21]	Cross-sectional study	338	To identify barriers to eye healthcare access and associated factors in adults through the 1E1F community outreach program	The primary barriers reported were hospital crowding (40.7%), financial constraints (30.0%), self-medication (20.5%), opting for traditional treatment (17.8%), and purchasing eyeglasses from street vendors (11.6%). Additional barriers included fear of treatment, waiting for conditions to worsen, perceived lack of necessity for care, distrust of professionals, time constraints, transportation issues, belief in the absence of solutions, limited knowledge, and lack of accompanying support	Areas for enhancing eye care access encompass interventions targeting underserved communities, investigating attitudes and practices related to eye care, promoting eye health education, expanding access to regular eye exams, implementing reminder systems for routine screenings, reducing service wait times at hospitals, and embracing digital health technologies such as telemedicine and AI to reach remote regions more effectively	Lack of representation of less-advantaged rural populations further from the city center of Nampula province
Thompson et al. (2015) [22]	Cross-sectional study	4601 selected in neighborhood clusters; 1087 with vision impairment	To understand barriers to accessing refractive services in the general population of Mozambique	The perceived cost of eye care and glasses emerged as the most significant barrier to accessing refractive services, cited by 53% of participants. Additionally, 28% reported not seeking treatment because they did not feel their problem was severe enough, while 15% identified distance to services as a barrier	Access can be enhanced by subsidizing care for the economically disadvantaged, through funds generated from treating wealthier patients. Other strategies include providing ready-made spectacles, intensifying eye health education, and advocating for eye care in rural regions	The study's methodology limited participants to selecting up to three barriers, hindering comprehensive ranking and analysis
Ezinne et al. (2023) [23]	Cross - sectional study	500	To determine the utilization of eye care services in an underserved community in Enugu State, Nigeria	Primary barriers to eye care utilization in the region included high costs (30%) and the overall unaffordability of insurance in Nigeria, distance to eye care facilities (22.6%), and inadequate road infrastructure (15.2%). Additionally, 30.6% of respondents sought alternative treatment methods due to issues related to proximity and affordability	These findings indicate the necessity for making eye care services affordable and accessible in this community, aiming to alleviate the impact of visual impairment and blindness	The study's findings are constrained by recall bias and the fact that it was conducted solely within the Ugbawka community, limiting its generalizability to the broader population.
Olusanya et al. (2016) [24]	Cross - sectional study	643	To describe the factors that determine the utilization of eye care services in a rural community in South-Western Nigeria	Only 19% of respondents had utilized orthodox eye care facilities previously. Factors increasing the likelihood of eye care utilization were similar, including age ≥70 years, literacy, proximity to eye care facilities, diabetes or hypertension, history of ocular symptoms, and blindness	Health education and awareness campaigns about the importance and benefits of seeking eye care early and the provision of community-based eye care programs	Inaccurate age estimates for illiterate participants, potential recall bias, and the focus solely on previous visits to orthodox facilities
Morka et al. (2020) [25]	Community-based cross-sectional study	668	To determine eye care service utilization and related factors among adults aged 40 years and above in Hawassa City, South Ethiopia, in June 2019	Eye care service utilization over the past two years was 23.8%. Factors positively linked to utilization included a history of eye disease, awareness of the importance of regular eye checkups, older age, and higher family monthly income	Recommended to provide eye health education for the community to increase awareness about eye care service utilization	Recall or social desirability biases in self-reported data and the absence of a temporal relationship between predictors and the outcome variable
Rono Mmed et al. (2019) [26]	Retrospective analysis	20,695	To investigate the key barriers to accessing eye care in Kenya	This study identified distance to facilities and gender as the main barriers to accessing eye care, with females facing challenges due to male dominance in household decision-making. Participants reported resorting to nonprescription drugs for eye issues, highlighting financial constraints in affording eye care expenses	The study suggests designing eye health services to enhance accessibility and alleviate the burden on secondary-level facilities. Specifically, it recommends improving secondary service capacity to ensure equitable access for individuals of all ages, genders, and distances from facilities	Incomplete records were noted, and the study did not include data on individuals who did not use public health services or sought treatment elsewhere
Aggarwal et al. (2018) [27]	Convenience sampling method/ survey	644	To investigate self-reported vision and access to vision services in Mwanza, Tanzania	Key factors hindering eye care utilization included cost concerns and perceptions about vision. Limited awareness of eye diseases was noted. Among employed respondents, vision problems affected work performance for 37%, leading to job discontinuation for 3.5% due to cross-sectional health issues such as anxiety and embarrassment	Lowering the expenses associated with vision care appointments could increase the utilization of vision healthcare services in Mwanza	The study's limitations include selection bias due to its focus on hospital patients, incomplete survey responses from many participants, and its exclusive concentration on adults aged 18 and above, potentially limiting its applicability to children with eye issues
Sukati et al. (2021) [28]	Cross-sectional study	243	To investigate the knowledge and practices of teachers about child eye health in the public sector	Many teachers recognize their schools admitting children with visual problems due to the lack of universal vision screening. Variations in travel methods and routes to healthcare facilities suggest disparities in access influenced by socioeconomic status and geography. The significance of healthy eyes for academic success and optimal classroom performance is underscored	Enhancing access to eye care involves training teachers on eye conditions to bridge knowledge gaps. Furthermore, collaboration between education and health ministries is crucial for effectively integrating eye health into school programs	Limitations include potential sampling and recall biases, focus solely on public health system service provision, and a small sample size
Sukati et al. (2018) [29]	Mixed methods review (qualitative & quantitative)	173	To assess the accessibility of child eye health services in Swaziland utilizing categories outlined by the World Health Organization framework	The Ministry of Health inadequately managed school health programs, with funds often misused or unaccounted for. - Swaziland experiences a significant shortage of eye care practitioners, and equipment, particularly in rural and less affluent areas, and lacks ophthalmologists specially trained in pediatric populations. Additionally, there is a lack of awareness among the general public and eye health professionals regarding the importance of child eye health and best practices for treating visual impairment and eye diseases	Enhancing access to eye care involves redistributing services to rural areas, offering pediatric care training for ophthalmologists, fostering partnerships between government, NGOs, and the private sector to expand access and offer financial aid, establishing a more efficient referral system linking rural communities with tertiary care, and providing comprehensive vision screening training for teachers to alleviate the workload of healthcare professionals	Small sample size and poor generalizability due to analysis of a specific population
Akuffo et al. (2024) [30]	Cross-sectional survey	213	To characterize practice patterns of low vision services among optometrists in Ghana	Over 70% of optometrists lacked assistive devices/ necessary equipment and eye examination kits, serving as a major barrier to providing eye care. Additionally, it was reported that lack of awareness of low vision centers, the high cost of low vision aids, and socially unacceptable assistive devices served as additional barriers/deterrents for patients seeking eye care	This study suggested public education campaigns, establishing low-vision centers, and making low-vision aids more affordable to encourage greater utilization of low-vision services among this demographic	The study's findings are limited by possible recall bias and the rural study setting, suggesting the need for cross-validation mechanisms in future surveys to enhance data reliability and generalizability
Trotignon et al. (2022) [31]	Cross - sectional study	1358	To evaluate the effectiveness of the coordinated approach To community health program, facilitated by sightsavers, in reaching the most impoverished individuals, women, and individuals with disabilities residing in Kasungu district, Malawi	Attendees at outreach camps exhibited significant poverty levels, though they were relatively wealthier compared to the national and Kasungu populations in 2011. Poverty rate comparisons from 2011 to 2017 showed that camp attendees were wealthier initially but became comparable to the Kasungu population by 2017. The study also observed a higher prevalence of disability among camp participants, highlighting potential community challenges. Women faced challenges accessing camps, underscoring the need for targeted community mobilization efforts	The study emphasized outreach camps' effectiveness in reaching economically disadvantaged individuals and those with disabilities. Tools like the Simple Poverty Scorecard, Equity Tool, and Washington Group Short Set of Questions on Disability were recommended for assessing equity in accessing eye care services	Monitoring equity in eye care programs was underscored, advocating for additional data on the patient's residence, clinical diagnoses, and referral uptake
Balarabe et al. (2014) [32]	Cross-sectional study	216	To determine the types of intervention sought by the blind street beggars and assess the barriers to accessing available eye care services	Among those seeking intervention, 18.8% went to hospitals, while others opted for self-medication (42.1%), medicine stores (31.2%), or traditional facilities (7.9%). Barriers to hospital treatment included unavailability of services and failure of relatives to transport blind individuals to health facilities	Provision of affordable, accessible, and qualitative eye care services with a strong health education component on avoidable causes of blindness	Limitations include poor generalizability due to analysis of only a specific population

Limitations

A limitation of this scoping review is the lack of a formal quality assessment or risk of bias assessment for the included studies.

## Conclusions

This scoping review found that a multitude of factors, including cost, education and cultural beliefs, and lack of facilities, act as barriers to access to eye care in SSA. In conclusion, addressing the challenges of eye care access in SSA necessitates intervention strategies that extend beyond the traditional confines of the healthcare sector. Recognizing the multifaceted nature of these challenges makes it apparent that collaboration with stakeholders in addressing broader livelihood issues is imperative. Initiatives aimed at improving food security, enhancing educational opportunities, and addressing SDOH must be integrated into comprehensive strategies for enhancing vision care accessibility. Only through such holistic approaches can sustainable improvements be achieved, ensuring that individuals across SSA have equitable access to quality eye care services. Therefore, prioritizing collaboration and integrating interventions across various sectors are essential steps toward achieving comprehensive and sustainable improvements in vision care accessibility throughout the region.
